# Assessment of Bones Deficient in Fibrillin-1 Microfibrils Reveals Pronounced Sex Differences

**DOI:** 10.3390/ijms20236059

**Published:** 2019-12-01

**Authors:** Lukas Altinbas, Nicole Bormann, Daniel Lehmann, Sarah Jeuthe, Dag Wulsten, Uwe Kornak, Peter N. Robinson, Britt Wildemann, Georgios Kararigas

**Affiliations:** 1 BIH Center for Regenerative Therapies, Charité – Universitätsmedizin Berlin, Corporate Member of Freie Universität Berlin, Humboldt-Universität zu Berlin, and Berlin Institute of Health, 13353 Berlin, Germany; 2 Julius Wolff Institute for Biomechanics and Musculoskeletal Regeneration, Charité – Universitätsmedizin Berlin, Corporate Member of Freie Universität Berlin, Humboldt-Universität zu Berlin, and Berlin Institute of Health, 13353 Berlin, Germany; 3 Max-Delbrück-Center for Molecular Medicine, 13125 Berlin, Germany; 4 Institute for Medical Genetics and Human Genetics, Charité – Universitätsmedizin Berlin, Corporate Member of Freie Universität Berlin, Humboldt-Universität zu Berlin, and Berlin Institute of Health, 13353 Berlin, Germany; 5 The Jackson Laboratory for Genomic Medicine, Farmington, CT 06032, USA; 6 Experimental Trauma Surgery, University Hospital Jena, 07743 Jena, Germany; 7 Charité – Universitätsmedizin Berlin, Corporate Member of Freie Universität Berlin, Humboldt-Universität zu Berlin, and Berlin Institute of Health, 10117 Berlin, Germany; 8 DZHK (German Centre for Cardiovascular Research), Partner Site Berlin, Germany

**Keywords:** biomechanics, bone architecture, fibrillin, Marfan syndrome, sex, TGFβ signaling

## Abstract

Defects in the extracellular matrix protein fibrillin-1 that perturb transforming growth factor beta (TGFβ) bioavailability lead to Marfan syndrome (MFS). MFS is an autosomal-dominant disorder, which is associated with connective tissue and skeletal defects, among others. To date, it is unclear how biological sex impacts the structural and functional properties of bone in MFS. The aim of this study was to investigate the effects of sex on bone microarchitecture and mechanical properties in mice with deficient fibrillin-1, a model of human MFS. Bones of 11-week-old male and female *Fbn1^mgR/mgR^* mice were investigated. Three-dimensional micro-computed tomography of femora and vertebrae revealed a lower ratio of trabecular bone volume to tissue volume, reduced trabecular number and thickness, and greater trabecular separation in females vs. males. Three-point bending of femora revealed significantly lower post-yield displacement and work-to-fracture in females vs. males. Mechanistically, we found higher Smad2 and ERK1/2 phosphorylation in females vs. males, demonstrating a greater activation of TGFβ signaling in females. In summary, the present findings show pronounced sex differences in the matrix and function of bones deficient in fibrillin-1 microfibrils. Consequently, sex-specific analysis of bone characteristics in patients with MFS may prove useful in improving the clinical management and life quality of these patients, through the development of sex-specific therapeutic approaches.

## 1. Introduction

Microfibrils and elastic fibers are the primary components of the architectural scaffold of connective tissue [1]. Fibrillin molecules—together with other extracellular matrix proteins—make up microfibrils and regulate microfibril biogenesis and function [2]. Gene defects in fibrillin-1 give rise to Marfan syndrome (MFS; OMIM #154700). MFS is an autosomal-dominant connective-tissue disorder, primarily characterized by musculoskeletal, ocular, pulmonary and cardiovascular anomalies, affecting about 2–3 in every 10,000 individuals [3,4]. The severity of clinical manifestations and the age of onset vary widely. Mutations in fibrillin-1 lead to severe skeletal abnormalities, including malformations of the limbs, spine and anterior chest wall [5]. Although reduced bone mass (osteopenia) is a controversial finding in MFS [2], decreases in bone mineral density (BMD) have been reported in adult patients [6,7,8,9] and children with MFS [9,10,11,12].

In many frequent pathological conditions, there are significant differences between men and women [13,14]. In the literature, we found only a few studies of adult patients with MFS that present data separately for each sex. In these limited reports, we observed that women appear to have, for example, lower BMD values than men [8,15]. However, whether this is true and a general phenomenon in MFS patients is not known, as any sex differences in MFS have not been systematically investigated.

Mice harboring a mutation in the *Fbn1* gene (*Fbn1^mgR/mgR^*) model a progressive form of human MFS [16]. MFS-associated skeletal anomalies have been documented in this and other mouse models of fibrillin-1 disruption [5]. In particular, *Fbn1^mgR/mgR^* mice develop osteopenia [17], i.e., reduced BMD. Although age-related changes in trabecular architecture differ between healthy male and female C57BL/6J mice [18], it is not understood whether there are any differences between male and female MFS mice in terms of bone structural and functional properties.

In the present study, in order to address this knowledge gap, we aimed at characterizing the bone microarchitecture and functional properties of male and female *Fbn1^mgR/mgR^* mice, hypothesizing pronounced sex differences. Considering that MFS is associated with transforming growth factor beta (TGFβ) over-activation [3,4,19], we also assessed the regulation of TGFβ signaling as a mechanistic factor contributing to sex-specific skeletal manifestations of MFS.

## 2. Results

### 2.1. Study Design and Body Mass

**Figure 1:**
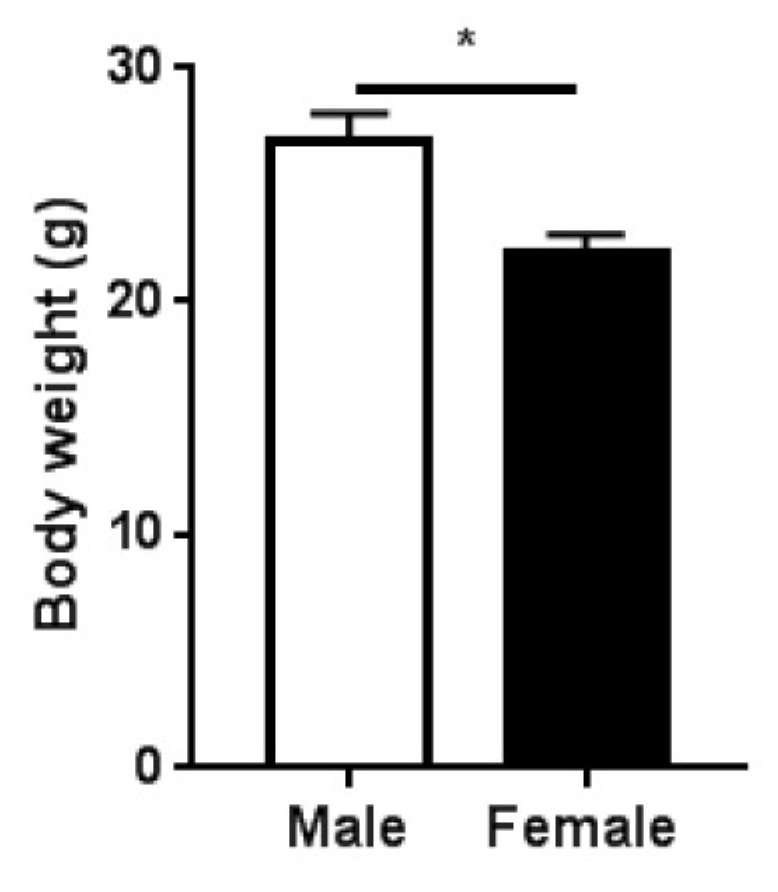
Body mass of male (*n* = 5) and female (*n* = 6) *Fbn1^mgR/mgR^* mice at the age of 11 weeks. Data present mean ± SEM; *p < 0.05.

In the present study, we sought to investigate the effects of sex on the bone microarchitecture and functional properties of adult mice with deficient fibrillin-1. For this purpose, we employed male and female *Fbn1^mgR/mgR^* mice, a model of human MFS, which have an average survival period of ca. 4 months [16]. Based on this, we initially planned to study eight male and eight female *Fbn1^mgR/mgR^* mice at the age of 11 weeks. During the course of the study, there were three deaths in the male group and two deaths in the female group. At 11 weeks of age, the female *Fbn1^mgR/mgR^* mice weighed significantly less than the male *Fbn1^mgR/mgR^* mice (Figure 1).

### 2.2. Bone Microarchitecture

**Figure 2:**
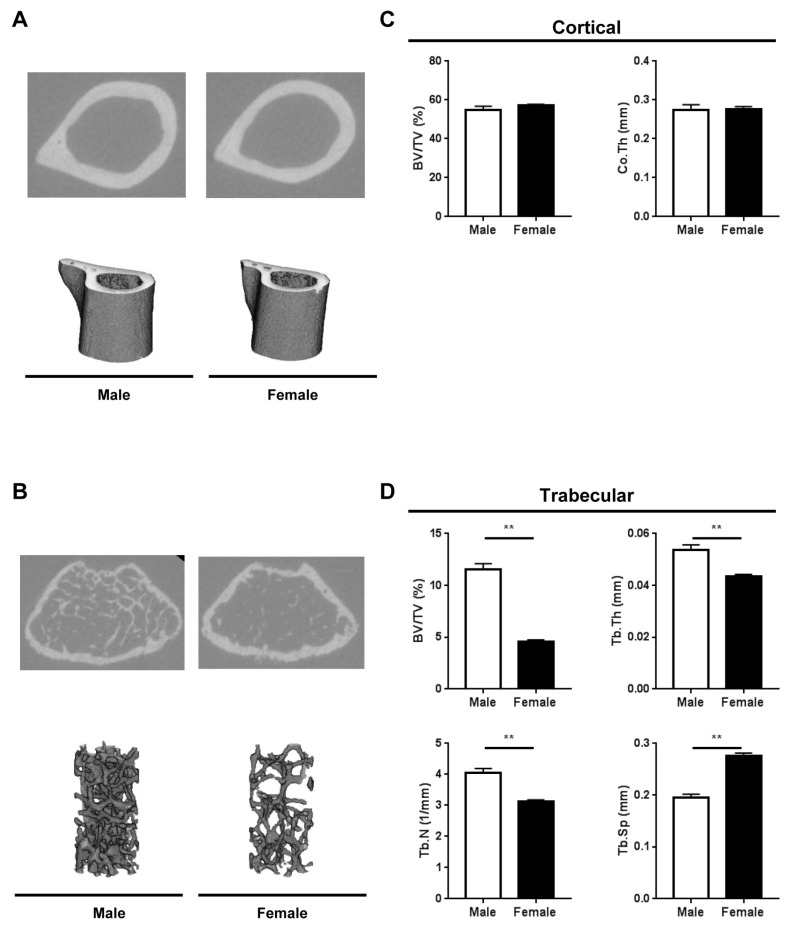
Cortical and trabecular microarchitecture of femora from male (*n* = 5) and female (*n* = 6) *Fbn1^mgR/mgR^* mice as determined by micro-computed tomography (μCT). (**A**) Representative μCT images of cortical bone cross sections (top) and 3D reconstructions showing cortical bone (bottom). (**B**) Representative μCT images of trabecular bone cross sections (top) and 3D reconstructions showing trabecular architecture (bottom). (**C**) Quantification of cortical parameters at the femoral diaphysis. (**D**) Quantification of trabecular parameters at the distal femoral metaphysis. BV/TV, % bone volume; Co.Th, cortical thickness; Tb.N, trabecular number; Tb.Sp, trabecular separation; Tb.Th, trabecular thickness. Data present mean ± SEM; **p < 0.01.

To determine the effects of sex on bone composition, we evaluated the microarchitecture of femora and vertebrae from male and female *Fbn1^mgR/mgR^* mice using micro-computed tomography (μCT). Our analysis revealed no significant differences between male and female femora in cortical bone parameters (Figure 2A,C). In contrast, there were significant sex differences in the trabecular microarchitecture of femora (Figure 2B,D). In particular, the trabecular bone volume fraction, thickness and number were significantly lower in female femora compared with male femora, while trabecular separation was significantly higher in female femora compared with male femora (Figure 2B,D).

**Figure 3:**
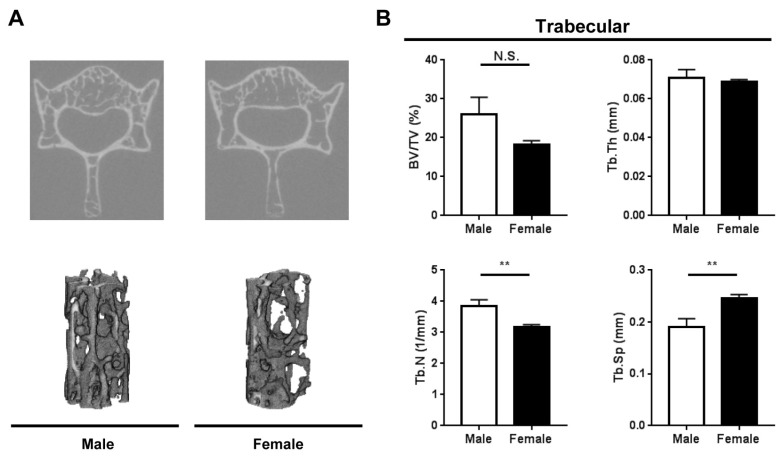
Trabecular microarchitecture of vertebrae from male (*n* = 5) and female (*n* = 6) *Fbn1^mgR/mgR^* mice as determined by μCT. (**A**) Representative μCT images of trabecular bone cross-sections (top), and 3D reconstructions showing trabecular architecture (bottom). (**B**) Quantification of trabecular parameters at the third lumbar vertebral body. BV/TV, % bone volume; N.S., not significant; Tb.N, trabecular number; Tb.Sp, trabecular separation; Tb.Th, trabecular thickness. Data present mean ± SEM; **p < 0.01.

Similarly to the femora, there were marked sex differences in the trabecular architecture of the vertebrae (Figure 3). There was a strong trend for lower trabecular bone volume fraction in female vertebrae compared with male vertebrae, which, however, did not reach statistical significance (*p* = 0.1). The trabecular bone number was significantly lower in female vertebrae compared with male vertebrae, while the trabecular separation was significantly higher in female vertebrae compared with male vertebrae (Figure 3). There was no difference in trabecular bone thickness.

### 2.3. Whole-Bone Mechanical Properties

**Figure 4:**
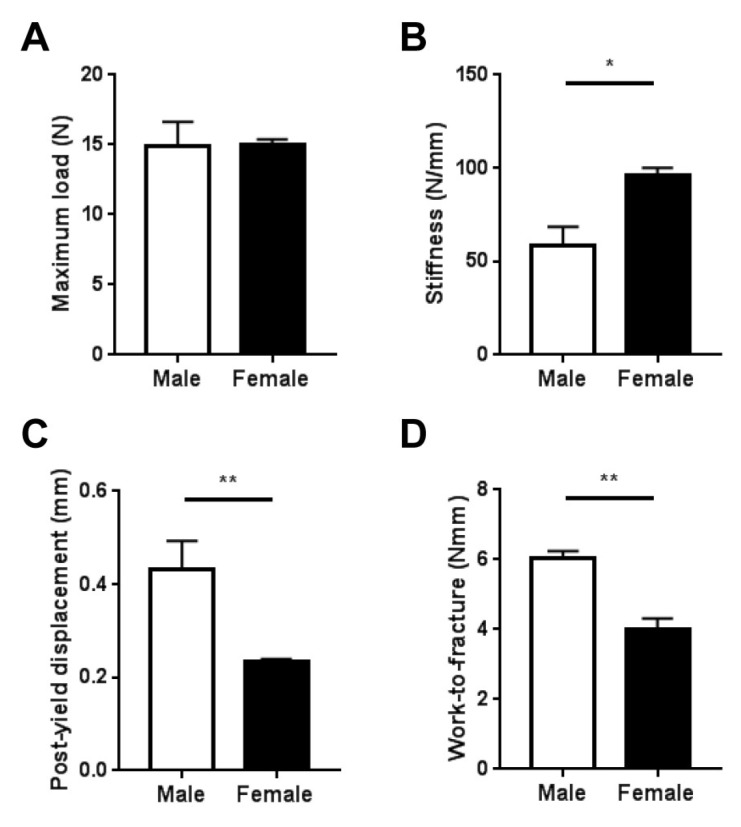
Bone biomechanical integrity. The whole-bone mechanical parameters maximum load (**A**), stiffness (**B**), post-yield displacement (**C**), and work-to-fracture (**D**) of femora from male (*n* = 5) and female (*n* = 6) *Fbn1^mgR/mgR^* mice are shown. Data present mean ± SEM; *p < 0.05, **p < 0.01.

To determine the effects of sex on mechanical properties, we subjected femora from male and female *Fbn1^mgR/mgR^* mice to biomechanical testing via 3-point bending. Our analysis revealed that the maximum load did not differ between male and female femora (Figure 4A). However, female femora had significantly greater stiffness compared with male femora (Figure 4B), while post-yield displacement and work-to-fracture were significantly lower in female femora compared with male femora (Figure 4C,D).

### 2.4. TGFβ Signaling

**Figure 5:**
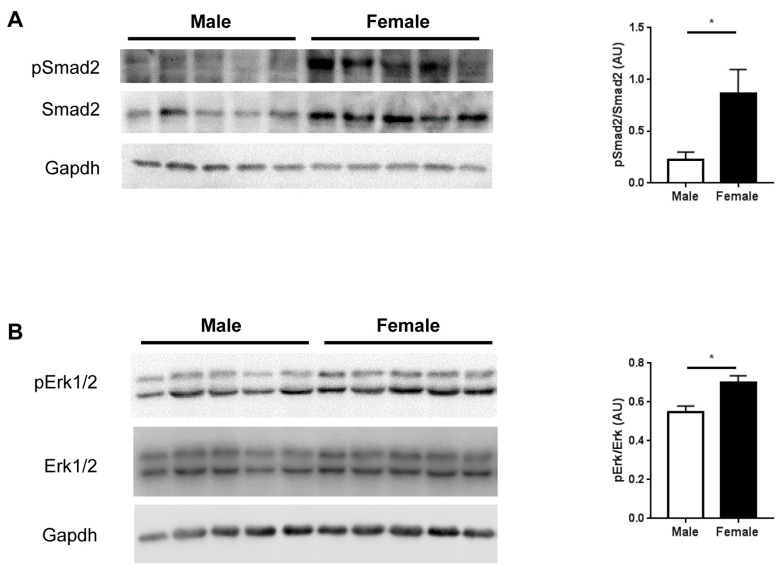
Assessment of transforming growth factor beta (TGFβ) pathway markers. The detection of phosphorylated and total Smad2 (**A**), and phosphorylated and total Erk1/2 (**B**), by immunoblotting with Gapdh as a loading control, as well as the corresponding quantification in tibiae from male (*n* = 5) and female (*n* = 5) *Fbn1^mgR/mgR^* mice, is shown. Data present mean ± SEM; *p < 0.05; AU, arbitrary unit.

To understand the underlying mechanisms contributing to differences in bone properties between male and female *Fbn1^mgR/mgR^* mice, we assessed the activation of TGFβ signaling by measuring the phosphorylation of Smad2 and Erk1/2, the relevance of which in MFS has been shown [3,4,19]. In line with our hypothesis of elevated TGFβ signaling in females vs. males, immunoblotting analysis revealed significantly higher levels of phosphorylated Smad2 and Erk1/2 in female tibiae compared with male tibiae (Figure 5).

## 3. Discussion

In the present study, we investigated the role of sex in microarchitecture and mechanical properties of bones at axial and appendicular sites in male and female mice harboring a mutation in the *Fbn1* gene (*Fbn1^mgR/mgR^*), a model of human MFS. To our knowledge, this is the first study reporting significant differences in trabecular bone and mechanical properties of femora between male and female *Fbn1^mgR/mgR^* mice. In addition, our biochemical analysis revealed significant sex differences that may contribute to the mechanisms impacting bone quality in a sex-specific manner.

Although there were no major sex differences in cortical bone microarchitecture parameters of femora, the trabecular bone phenotype differed significantly between male and female *Fbn1^mgR/mgR^* mice. In particular, compared with male *Fbn1^mgR/mgR^* mice, female *Fbn1^mgR/mgR^* mice displayed significant reductions in trabecular bone volume fraction, thickness and trabecular number, as well as a significant increase in trabecular separation. Sex differences in trabecular architecture at the vertebrae followed similar patterns as the femora. Together, these data indicate that there are pronounced sex differences in metabolic signals among osteocytes and osteoclasts, leading to sex-specific bone mass phenotypes.

Our whole-bone biomechanical analysis revealed that there were no sex differences in the maximum load before fracture. However, stiffness was significantly higher in female femora than in male femora, while post-yield displacement was significantly lower in female femora than in male femora. Along this line, the overall resistance to failure—as assessed by work-to-fracture—was significantly lower in female femora compared with male femora. These findings indicate that female femora present brittle behavior that may be more prone to fracture.

To elucidate underlying mechanisms contributing to sex-specific bone remodeling in *Fbn1^mgR/mgR^* mice, we verified the activation of TGFβ signaling, assessing the phosphorylation levels of Smad2 and Erk1/2. Our analysis revealed that the levels of phosphorylated Smad2 and Erk1/2 were significantly higher in female tibiae when compared with male tibiae. Normal fibrillin-1, which forms extracellular microfibrils, is involved in regulating the bioavailability of TGFβ [20]. However, defects in fibrillin-1 in MFS disturb this regulatory mechanism, causing the activation of TGFβ signaling, which, in turn, leads to the phosphorylation of the downstream signal transducers Smad2 and Erk1/2 [21,22]. Notably, decreased bone volume and density in *Fbn1^mgR/mgR^* mice was reported to be due to increased osteoclastogenesis, which was largely attributed to the activation of TGFβ signaling [17]. Therefore, the present findings indicate that there is a greater activation of TGFβ signaling in female vs. male *Fbn1^mgR/mgR^* mice, which may underlie the sex differences in microarchitecture and mechanical properties of bones in this model of human MFS.

Our analysis was limited to one age (11 weeks), and to femora, tibiae and vertebrae. Therefore, these data may not extrapolate to animals of other ages or other parts of the skeleton. However, the comparisons presented here demonstrate, for the first time, a role of sex in microarchitecture and mechanical properties of bones at axial and appendicular sites in *Fbn1^mgR/mgR^* mice. The major driver of the sex differences reported here is also not clear. We speculate that estrogen may play an important role, and it would be worth examining these findings in estrogen-depleted and -treated animals to test this hypothesis. In addition, considering previous findings in relation to the heart—where the response to treatment may differ significantly between the sexes [23,24] or between different genetic backgrounds [25]—and the contribution of multiple genetic variations to disease development and drug responses [26,27], it would be of interest to determine the role of sex in bone remodeling using different animal models of MFS. Furthermore, although the role of aberrant TGFβ signaling in the pathophysiology of MFS is well established, there is an incomplete understanding of the contributing mechanisms [28], particularly in MFS-associated skeletal pathophysiology. Moreover, the objective of this analysis was focused on comparing male and female *Fbn1^mgR/mgR^* mice directly and not with wild-type mice. Therefore, it cannot be excluded that some of the differences observed here might reflect physiological skeletal differences between males and females as described previously for healthy C57BL/6J mice [18]. Further research is therefore warranted.

## 4. Materials and Methods 

### 4.1. Experimental Animals

Male and female *Fbn1^mgR/mgR^* (*n* = 16) mice were employed and genotyped as described previously [16]. The heterozygous mutant progeny was originally provided by Dr. Francesco Ramirez and the animals analyzed in this study come from a colony held on our site [29]. The mice were kept on a 12–12 h light–dark cycle in temperature-controlled rooms with water ad libitum. At 11 weeks of age, the mice were euthanized and the long bones—the femora and tibiae—as well as the vertebrae, were collected, stripped of soft tissues and prepared for μCT, or wrapped in saline-soaked gauze and frozen at −20 °C (for biomechanical testing), or snap frozen in liquid nitrogen and stored at −80 °C (for immunoblotting), until further analyses were performed. All experiments were performed following the guidelines of the EU Directive 2010/63/EU for animal experiments, and were approved by the Landesamt für Gesundheit und Soziales, Berlin, Germany (Nr. G0090/14, 01.08.2014).

### 4.2. Micro-Computed Tomography

Three-dimensional micro-computed tomography (μCT) analysis was performed on femora and vertebrae. One femur and the third lumbar vertebral body from each animal were scanned using the vivaCT40 scanner (SCANCO Medical AG, Brüttisellen, Switzerland). For all scans, a resolution of 10.5 µm, a voltage of 70 kV, a current of 114 µA, and a 0.5 mm aluminum filter were used. The scans were analyzed with Fiji (ImageJ, Bethesda, MD, USA [30]). Image stacks were aligned using landmarks and the volume of interest (VOI) was set. For trabecular bone parameters, the starting point of the VOI was set at the distal femur, directly at the end of the growth plate, with a diameter of 0.75 mm and a height of 1.5 mm. To analyze cortical parameters of the femur diaphysis, the VOI started 5 mm proximally of the VOI for trabecular bone, with a height of 5 mm including the complete cortical area. The VOI for the vertebrae was the same defined cylinder as for the trabecular bone of the femur and was set exactly in the middle of the third lumbar vertebral body.

### 4.3. Biomechanical Measurement

The biomechanical whole-bone strength was analyzed in destructive three-point bending experiments, using a Bose Test Bench LM 1 ElectroForce (Bose, Eden Prairie, MN, USA). Femora were mounted anterior side up for bending tests at an 8.5 mm span width between the end supports. The load was applied to the anterior midshaft of the femora at a constant deflection rate of 0.1 mm/s. Load (50 lbs/225 N load cell) and displacement data were acquired at 100 Hz. Stiffness, load, deflection and work were calculated from the force-deflection curve using a routine written in MATLAB (The Mathworks Inc., Natick, MA, USA). Whole-bone mechanical properties were adjusted for body mass using the linear regression method, according to the guidelines described previously [31].

### 4.4. Immunoblotting

Protein lysates were isolated from tibiae as described previously [32,33,34]. Independent biological replicates for each group were run separately on SDS-PAGE gels and transferred to nitrocellulose membranes using standard procedures. Primary antibodies against pSmad2, Smad2, pErk1/2, Erk1/2 (all from Cell Signaling Europe, Frankfurt am Main, Germany), and Gapdh (Thermo Fisher, Darmstadt, Germany; loading control) were used. For immunodetection, secondary antibody donkey anti-rabbit or anti-mouse (Dianova, Hamburg, Germany) and ECL™ Prime Western Blotting Reagent (Amersham, Munich, Germany) were used. Data were quantified with the ImageJ software version 1.52a (http://rsbweb.nih.gov/ij/).

### 4.5. Statistical Analysis

All data are presented as mean ± SEM. Statistical significance was assessed using PRISM version 7 (GraphPad Software Inc., San Diego, CA, USA). Comparisons between two groups were made with non-parametric Mann–Whitney *U* tests. *p* ≤ 0.05 was considered significant.

## 5. Conclusions

Whether there are any sex differences in bone mass and matrix properties of human MFS patients is not known. The present study shows that the bone microarchitecture and functional properties of mice with deficient fibrillin-1 differ significantly between males and females. Consequently, these findings call for a sex-specific analysis of bone characteristics (and fractures) in patients with MFS. As no cure is available, current treatment strategies target the improvement of patients’ symptoms through pharmacological approaches, to tackle primarily pulmonary and cardiovascular phenotypes. However, there are studies indicating that not all MFS patients may respond to such treatments, which may also not be effective against some manifestations of MFS, such as those affecting the skeleton [35,36,37]. Given that there may be pronounced sex differences in responses to pharmacological interventions [27], it is necessary to determine the role of sex in standard treatment strategies used with MFS patients. Artificial intelligence, by means of in silico models, may be useful in predicting sex-specific drug responses, thereby facilitating their translation into clinical practice [38,39]. Lastly, due to the clear need for the development of novel and effective strategies addressing the skeletal abnormalities in MFS, we put forward that sex-specific therapies would contribute to more appropriate and personalized medical care, thereby addressing the needs of male and female patients.

## Abbreviations

BMD	bone mineral density
MFS	Marfan syndrome
μCT	micro-computed tomography
TGFβ	transforming growth factor beta
